# Suicide and self-harm in women with mental disorders during pregnancy and the year after birth

**DOI:** 10.1192/j.eurpsy.2021.459

**Published:** 2021-08-13

**Authors:** K. Ayre, A. Bittar, R. Dutta, L. Howard

**Affiliations:** 1 Health Service And Population Research; Institute Of Psychiatry, Psychology And Neuroscience, King’s College London, SE AF, United Kingdom; 2 Institute Of Psychiatry, Psychology And Neuroscience, Academic Department of Psychological Medicine, RJ, United Kingdom

**Keywords:** Suicide, self-harm, perinatal, women’s mental health

## Abstract

**Introduction:**

There is little prospective data on the risk factors for later suicide in women who experience perinatal mental disorders, particularly beyond one-year postnatal.

**Objectives:**

Among a cohort of women who were in contact with a mental healthcare provider during the perinatal period, to: (1) Describe sociodemographic and clinical characteristics of the women who died by suicide (2) Understand when, in relation to childbirth, most suicides tended to occur.

**Methods:**

Data-linkage of de-identified service-user electronic healthcare records, national hospital episode statistics and mortality data generated a cohort of women in contact with a mental healthcare service provider in London, UK, perinatally. Using Natural Language Processing and structured field extraction, we identified clinical, socio-demographic characteristics, self-harm exposure, and suicide.

**Results:**

Among 5204 women, clinical and demographic characteristics of women who did and did not die by suicide were similar apart from indicators of illness severity including perinatal sedative medication prescription, clinician-rated functional impairment and smoking, which were more common in women who died by suicide. Suicide deaths occurred most frequently in the second year post-delivery. The most common method of suicide ocurring wihtin two years was by violent means, whereas after two years postnatal, the most common method was non-violent.

**Conclusions:**

Our findings support the extension of perinatal mental healthcare service provision to two years post-delivery.

**Disclosure:**

No significant relationships.

